# Developing an Immune-Related Signature for Predicting Survival Rate and the Response to Immune Checkpoint Inhibitors in Patients With Glioma

**DOI:** 10.3389/fgene.2022.899125

**Published:** 2022-06-02

**Authors:** Sibin Zhang, Xu Xiao, Yu Wang, Tianjun Song, Chenlong Li, Hongbo Bao, Qing Liu, Guiyin Sun, Xiaoyang Sun, Tianqi Su, Tianjiao Fu, Yujie Wang, Peng Liang

**Affiliations:** ^1^ Department of Neurosurgery, Harbin Medical University Cancer Hospital, Harbin, China; ^2^ Department of Esophageal Surgery, Harbin Medical University Cancer Hospital, Harbin, China; ^3^ Department of Medicine II, University Hospital LMU Munich, Munich, Germany

**Keywords:** glioma, bioinformatics signature, prognosis, immune microenvironment, immunotherapy

## Abstract

**Background:** Glioma is one of the most aggressive cancer types affecting the central nerve system, with poor overall survival (OS) rates. The present study aimed to construct a novel immune-related signature to predict prognosis and the efficiency of immunotherapy in patients with glioma.

**Methods:** The mRNA expression data and other clinical information of patients with glioblastoma multiforme (GBM) and low grade glioma (LGG) were obtained from The Cancer Genome Atlas and Chinese Glioma Genome Atlas databases. The immune-related genes were obtained from the Immunology Database and Analysis Portal database. Subsequently, an immune-related signature was created following the results obtained from the Least Absolute Shrinkage and Selection Operator regression model. To validate the predictability of the signature, Kaplan-Meier survival curves and time-dependent receiver operating characteristic curves were created. Moreover, both univariate and multivariate analyses were carried out using the OS between this signature and other clinicopathologic factors, and a nomogram was constructed. In addition, the association between signature, immune cell infiltration, tumor mutation burden and immunophenoscore were determined.

**Results:** Results of the present study using 118 GBM and LGG samples uncovered 15 immune-related genes that were also differently expressed in glioma samples. These were subsequently used to construct the immune-related signature. This signature exhibits the ability to predict prognosis, the infiltration of immune cells in the tumor microenvironment and the response of patients with glioma to immunotherapy.

**Conclusion:** Results of the present study demonstrated that the aforementioned novel immune-related signature may accurately predict prognosis and the response of patients with glioma to immunotherapy.

## Introduction

Glioma is one of the most aggressive and common malignant cancer types affecting the central nerve system (CNS) (Chen et al., 2021a), (Wang et al., 2021), which poses a serious threat to human health worldwide. In elderly patients (age, >65 years), the 5-year survival rate is ∼5% (Hendriks et al., 2019). According to the 2021 World Health Organization classification standard, gliomas are divided into grades, namely I-IV (Louis et al., 2021). Notably, grade I glioma exhibits slow proliferation and an improved prognosis, and grade IV glioma (also known as glioblastoma multiforme; GBM) is highly proliferative and invasive, exhibiting rapid recurrence and poor rates of overall survival (OS) and progression-free survival (PFS) (Soeda et al., 2015; Karim et al., 2016). Despite the development of GBM therapy over numerous years, the prognosis of patients with GBM remains poor (Janjua et al., 2021). In patients with GBM, the OS and PFS are ∼14 and 7 months, respectively (Ostrom et al., 2014; Kelly et al., 2017). The standard therapy used for the treatment of GBM is surgery combined with radiotherapy and chemotherapy (Di Cintio et al., 2020). However, the current therapy model cannot prevent the recurrence of GBM effectively. As a result of these factors, a novel model to predict prognosis and novel therapy options for patients with glioma are required.

Following technological advances and progress made in immunotherapies, advances have been made in the treatment of multiple cancer types, including brain metastasis (Tawbi et al., 2018; Hendriks et al., 2019). Notably, there have been two main obstacles preventing the development of effective immunotherapies for GBM: 1) Development of a highly immunosuppressive microenvironment and 2) high tumor heterogeneity (Lim et al., 2018). However, a number of recent studies have suggested that anti-tumor responses to immunotherapy may also occur in the micro-environment in the brain, which may be promising for the development of novel treatment options for malignant gliomas (Li et al., 2021; Xun et al., 2021). Three types of immune checkpoint inhibitors (ICIs) have been tested to treat GBM; namely, anti-PD-1 antibody, anti-CTLA-4 antibody and anti-LAG-3 antibody (Preusser et al., 2015). Although the results of previous studies have suggested that the response rates of ICIs for glioma were not optimal (Berghoff et al., 2015; Kurz et al., 2018), these developments are useful to determine the reasons for therapy failure and to distinguish which patients with glioma may benefit from the current immunotherapy options. The tumor immune microenvironment (TIME) and immune-related genes (IRGs) play important roles in immunotherapy (Remon et al., 2021), and can be used as biomarkers for predicting the survival rates of patients (Pan et al., 2020) {Wan, 2021 #6}. However, few studies have previously explored whether IRGs and TIME may act as biomarkers for survival rate prediction and determining the response to immunotherapy in patients with glioma.

The present study aimed to create a novel signature for predicting both the prognosis and response to immunotherapy in patients with glioma, according to IRGs and TIME data obtained from public databases. Following the development of this signature, the association between clinicopathological factors and prognosis in glioma was also detected. Furthermore, the association between immune cell infiltration, tumor mutation burden (TMB), immunophenoscore (IPS) and this signature was determined in patients with glioma. Results of the present study demonstrated that the signature may aid in improved prognosis prediction, and may provide a novel theoretical basis for further elucidating immunotherapy options for glioma.

## Materials and Methods

### Expression and Clinical Information of Patients

Clinical characteristics and mRNA expression levels of GBM and LGG samples were obtained from The Cancer Genome Atlas (TCGA) database (https://portal.gdc.cancer.gov/) and the Chinese Glioma Genome Atlas (CGGA) database (http://www.cgga.org.cn/). After matching the expression information with the clinical information, a total of 1,008 samples were used in the present study, which included 1,003 tumor and 5 healthy samples.

### Differentially Expressed Immune-Related Genes (DEIRGs)

**FIGURE 1:**
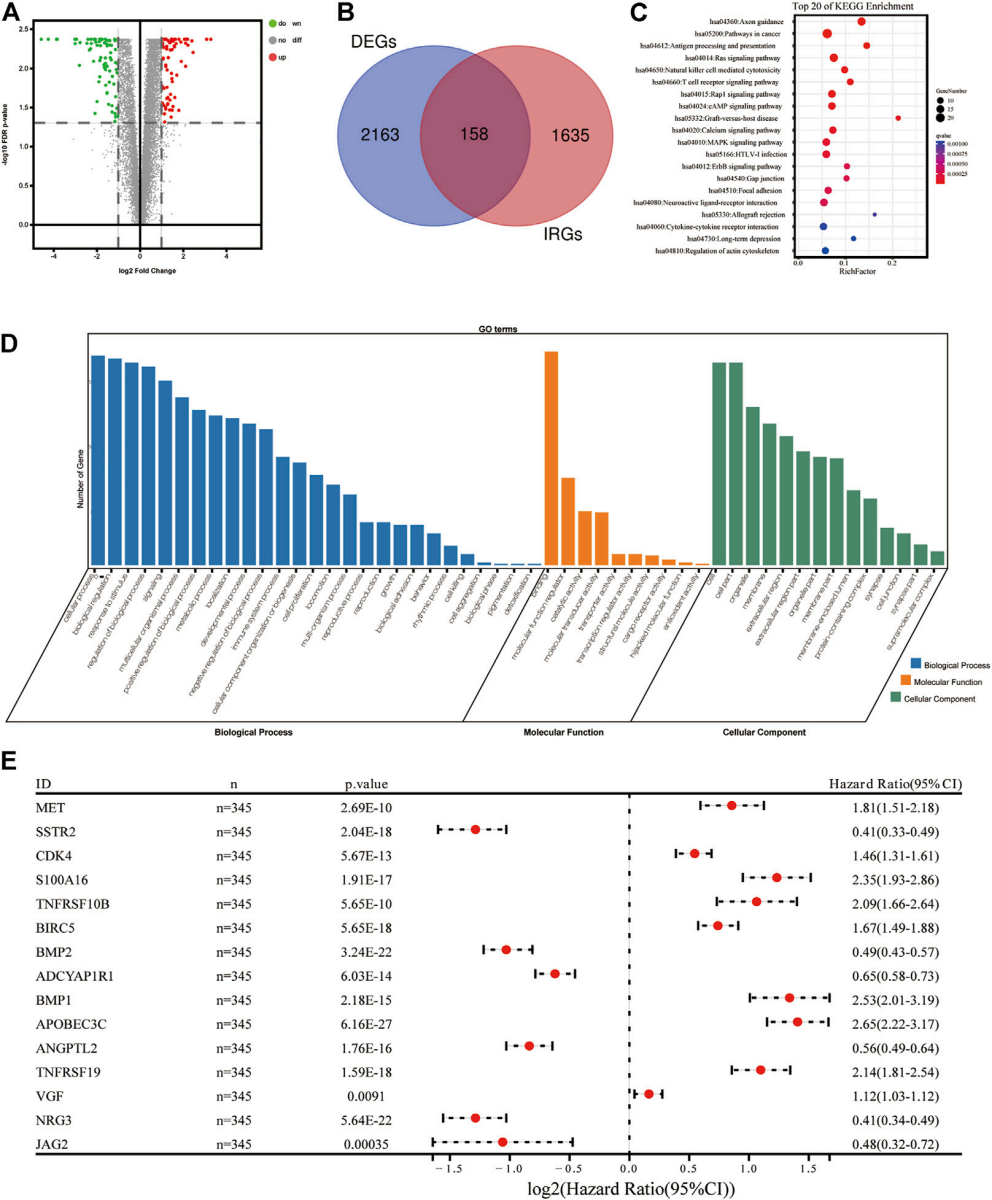
Construction of immune-related signature for glioma. **(A)** Volcano diagram of DEIRGs between glioma and healthy brain tissues. **(B)** Venn diagram demonstrating the intersected genes between DEGs and IRGs. **(C)** Top 20 biological progresses of KEGG enrichment analysis of 118 DEIRGs. **(D)** GO enrichment analysis of 118 DEIRGs. Blue, orange and green columns indicated Biological Process, Molecular Function, and Cellular Component, respectively. **(E)** Forest diagram demonstrating the multivariable cox model results of 15 hub genes in the immune-related signature. DEIRGS, differentially expressed immune-related genes; DEGs, differently expressed genes; IRGs, immune-related genes; KEGG, Kyoto Encyclopedia of Genes and Genomes; GO, Gene Ontology.

A total of 1,793 IRGs were downloaded from the Immunology Database and Analysis Portal (ImmPort) database (https://www.immport.org/home). Using the R Package “limma”, a total of 2,321 differently expressed genes (DEGs) were determined using the transcription information of 690 samples, which followed the thresholds (|log2(Fold Change)|>1 and false discovery rate (FDR) < 0.05. A total of 158 genes were extracted as DEIRGs, both existing in IRGs (1,793 genes) and DEGs (2321 genes); this process was visualized using a Venn diagram (Figure 1B). In the DEIRGs group, the expression levels of 80 genes were upregulated and 78 genes were downregulated. The volcano plot of DEIRGs was created using the R package “ggplot2”.

### Functional Enrichment Analyses

Two types of functional enrichment analyses, Gene Ontology (GO) and Kyoto Encyclopedia of Genes and Genomes (KEGG), were carried out to determine the biological functions of DEIRGs, using the online tool Database for Annotation, Visualization, and Integrated Discovery (DAVID) (version, 6.8; https://david.ncifcrf.gov/). The enrichment of GO terms and KEGG signaling pathways followed the threshold (FDR<0.05). Whole GO terms and the top 20 most significant KEGG pathways were visualized using the R package “ggplot2” (Figures 1C,D).

### Development and Accuracy Evaluation of the Immune-Related Signature

The 690 tumor samples obtained from the TCGA database were allocated into two sets randomly, named the TCGA Training Set (*n* = 345) and the TCGA Validation Set (*n* = 345). Moreover, the 313 tumor samples obtained from the CGGA database were named the CGGA Set (*n* = 313). All sample details are displayed in Table 1. The TCGA Training Set was used to construct the risk signature. TCGA Testing Set, Entire TCGA set and CGGA Set were applied to verify the prognostic accuracy. A univariate Cox proportional hazard regression analysis was carried out to select a total of 118 DEIRGs from the whole DEIRGs cohort, which associated with the OS of patients with glioma (*p* < 0.05; Supplementary Table S1). A least absolute shrinkage and selection operator (LASSO) test was performed on the 118 DEIRGs to minimize the error rate using the “glmnet” R package, and 15 hub genes were used to construct the prognostic signature of patients with glioma (Supplementary Figure S1). The formula of risk score was as followed: Risk score = [Expression level of Gene 1×coefcient]+[Expression level of Gene 2×coefcient]+…+[Expression level of Gene *n* × coefficient]. The risk scores from patients were calculated using the aforementioned formula. Patients were subsequently allocated into high-risk and low-risk groups by the median of risk scores. To verify the accuracy of this 15-gene risk signature, the K-M survival curves and receiver operating characteristic (ROC) curves were used to compare the survival rates of patients in the low-risk and high-risk groups using the “survival” and “survivalROC” R package.

**TABLE 1:** Clinical information of the entire TCGA set, TCGA training set, TCGA validation set and CGGA set. NA, Not available; TCGA, The Cancer Genome Atlas; CGGA, Chinese Glioma Genome Atlas.

Variables	Group	Entire TCGA set (*n* = 690)	TCGA training set (*n* = 345)	TCGA validation set (*n* = 345)	CCGA set (*n* = 313)
Survival time (days)		861 ± 34.95	858 ± 50.29	865 ± 48.62	1451 ± 83.24
Vital status	Alive (0)	416 (60%)	215 (62%)	201 (58%)	95 (30%)
	Dead (1)	274 (40%)	130 (38%)	144 (42%)	218 (70%)
Gender	Female	296 (43%)	158 (46%)	138 (40%)	116 (37%)
	Male	394 (57%)	187 (54%)	207 (60%)	197 (63%)
Age	≤65	601 (87%)	296 (86%)	305 (88%)	306 (98%)
	>65	89 (13%)	49 (14%)	40 (12%)	7 (2%)
Histological-type	Astrocytoma	194 (28%)	92 (27%)	102 (30%)	—
	Oligoastrocytoma	130 (19%)	69 (20%)	61 (18%)	—
	Oligodendroglioma	199 (29%)	101 (29%)	98 (28%)	—
	Glioblastoma	167 (24%)	83 (24%)	84 (24%)	—
Tumor-Grade	G2	257 (37%)	131 (38%)	126 (37%)	98 (31%)
	G3	266 (39%)	131 (38%)	135 (39%)	74 (24%)
	G4	167 (24%)	83 (24%)	84 (24%)	137 (44%)
	NA	—	—	—	4 (1%)
IDH-mutation-status	Wildtype	232 (34%)	111 (32%)	121 (35%)	145 (46%)
	Mutant	417 (60%)	215 (62%)	202 (59%)	167 (53%)
	NA	41 (6%)	19 (6%)	22 (6%)	1 (1%)
1p19q-co-deletion-status	Non-codel	486 (70%)	237 (69%)	249 (72%)	243 (78%)
	Codel	165 (24%)	91 (26%)	74 (21%)	62 (20%)
	NA	39 (6%)	17 (5%)	22 (6%)	8 (2%)
MGMTp-methylation-status	Methylated	—	—	—	152 (49%)
	Un-methylated	—	—	—	143 (46%)
	NA	—	—	—	18 (5%)

### Evaluation of Immune Cell Infiltration

Details of immune cell infiltration were obtained from the TCGA RNA-sequencing database and were calculated using the online tool CIBERSORT (https://cibersort.stanford.edu/).

### Analyses of IPS and TMB

IPS data were obtained from The Cancer Immunome Atlas (TCIA) (https://tcia.at/home). TMB data were obtained from the TCGA database (https://portal.gdc.cancer.gov/) and stored in Mutation Annotation Format (MAF). TMB analyses were carried out using the R package “maftools” and “TCGAmutations”.

### Statistical Analysis

Univariate and multivariate Cox regression calculations were applied using the R package “survival” to validate the predictability of the signature and clinicopathological factors. Differences between clinical features were determined using the independent t-test. *p* < 0.05 was considered to indicate a statistically significant difference.

## Results

### Construction of the Immune-Related Signature

According to the criteria (FDR<0.05 and |log2 (fold change)|>1), a total of 2,163 DEGs (811 upregulated and 1,352 downregulated genes) were identified and used in subsequent analyses. A Venn diagram was created to extract 158 DEIRGs (80 upregulated and 78 downregulated genes) from the DEGs cohort (Figures 1A,B). Results of the KEGG functional enrichment analysis demonstrated that DEIRGs exhibited a high significant correlation with the “Axon guidance” signaling pathway (Figure 1C). Moreover, results of the GO enrichment analysis suggested that “cellular process”, “binding” and “cell” were the most enriched terms of “BP”, “MF” and “CC”, respectively (Figure 1D).

To determine which genes demonstrated levels of correlation with prognoses of glioma, a univariate Cox regression analysis was carried out using 158 DEIRGs. Subsequently, results of the present study demonstrated that a total of 118 DEIRGs were associated with the OS of patients with glioma (*p* < 0.05). The 118 DEIRGs were subsequently used to perform the LASSO analysis for minimizing over-fitting rate, and 15 hub genes were applied to construct the immune-related signature (Figure 1E, Table 2). The risk score was calculated using the linear combination of the expression levels of the 15 hub genes, weighted by their relative coefficient in multivariate Cox regression as follows: Risk score = (0.210800714966123*MET)+(-0.031453990525702*SSTR2)+(0.16445568352528*CDK4)+(0.155012190659808*S100A16)+(0.0172907860024853*TNFRSF10B)+(0.0343382829300903*BIRC5)+(-0.166374735936659*BMP2)+(-0.0107626429700564*ADCYAP1R1)+(0.0595537300500025*BMP1)+(0.383363915934444*APOBEC3C)+(-0.127774932858095*ANGPTL2)+(0.0483829995201501*TNFRSF19)+(0.0311096000176334*VGF)+(-0.112719694494515*NRG3)+(-0.152886825995057*JAG2).

**TABLE 2:** Coefficients and multivariable Cox model results of 15 genes in the immune-related signature.

Gene	Log FC	Regulation	Coefficient	HR	Low 95%CI	High 95%CI	*p* Value
MET	−1.82	Down	0.2108	1.45336	1.14905	1.83827	0.00181
SSTR2	−1.52	Down	−0.03145	0.88981	0.67462	1.17363	0.40851
VGF	−2.8	Down	0.03111	1.14257	1.03617	1.25989	0.00753
NRG3	−1.27	Down	−0.11272	1.02575	0.73702	1.42757	0.8802
JAG2	−1.12	Down	−0.15289	0.61799	0.40754	0.9371	0.02346
CDK4	2.06	UP	0.16446	1.29865	1.13354	1.4878	0.00017
S100A16	1.26	UP	0.15501	1.30697	1.03726	1.64681	0.0232
TNFRSF10B	1.07	UP	0.01729	1.09709	0.80409	1.49687	0.55886
BIRC5	1.90	UP	0.03434	1.08989	0.88827	1.33727	0.4095
BMP2	2.12	UP	−0.16637	0.80824	0.63798	1.02395	0.07775
ADCYAP1R1	1.38	UP	−0.01076	0.94352	0.76966	1.15667	0.57588
BMP1	1.22	UP	0.05955	1.08548	0.73657	1.59967	0.67844
APOBEC3C	1.42	UP	0.38336	1.38023	1.03907	1.83342	0.02611
ANGPTL2	2.41	UP	−0.12777	0.81093	0.63773	1.03117	0.08734
TNFRSF19	1.81	UP	0.04838	1.00217	0.7706	1.30334	0.98708

### Immune-Related Signature and the Survival of Patients With Glioma

**FIGURE 2:**
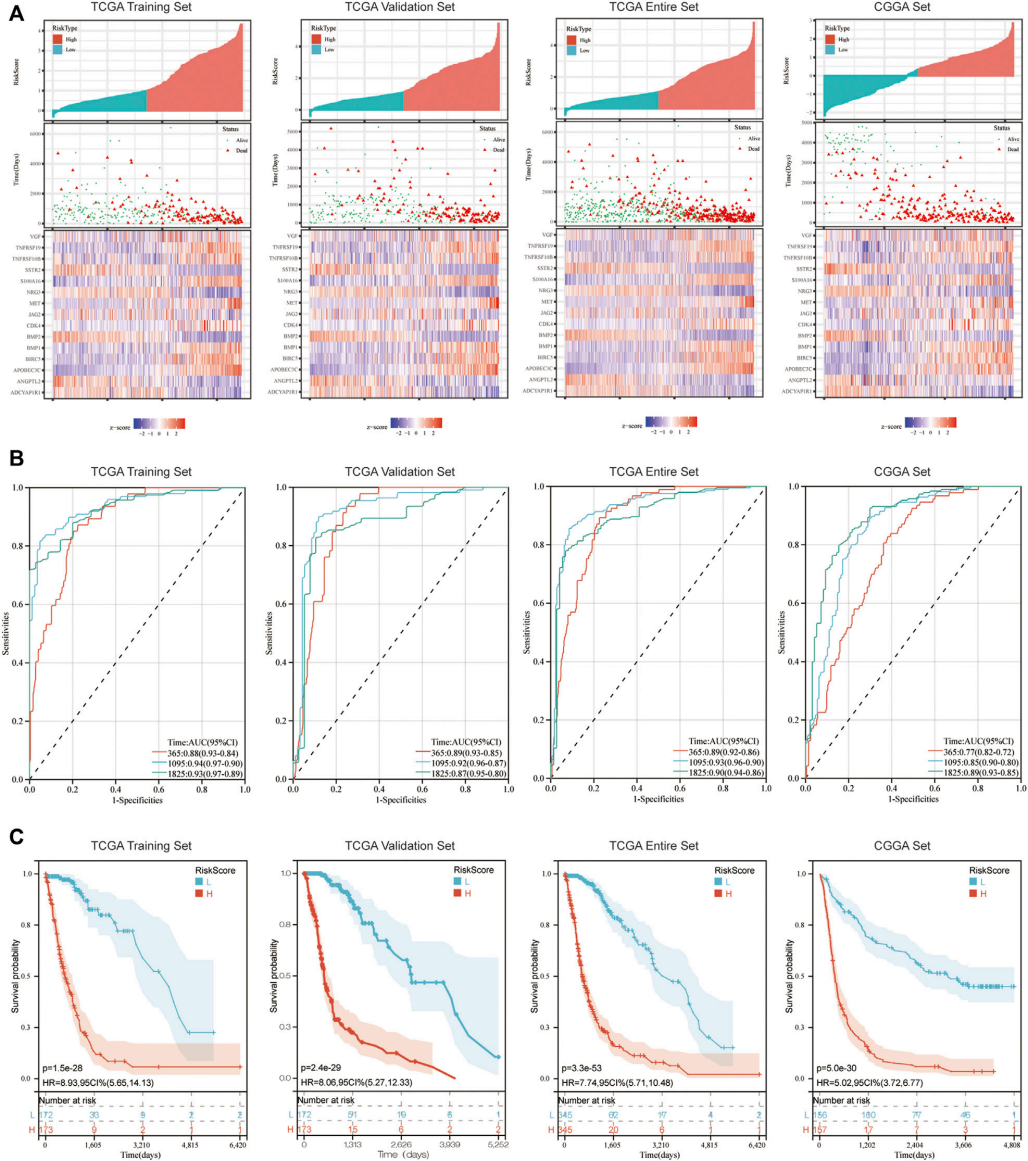
Immune-related signature effectively predicts the prognosis of patients with glioma. **(A)** Risk score distribution, survival status and expression of 15 hub genes for glioma in low-risk and high-risk groups. **(B)** 1, 3 and 5-year ROC curve analyses. ROC, receiver operating characteristic. **(C)** K-M survival curve analyses.

Patients were allocated into high-risk and low-risk groups according to the median of risk scores (Figure 2A). Results of the present study demonstrated that patients in the low-risk group exhibited a prolonged OS, compared with those in the high-risk group (*p* < 0.05; Figure 2C). Moreover, the accuracy of the immune-related signature was tested using time-dependent ROC curves (Figure 2B). In the TCGA training set, the under areas of 1-year, 3-year and 5-year survival were 0.88, 0.94 and 0.93, respectively, which demonstrated the ability of the immune-related signature to predict the prognosis of patients with glioma. The same ROC curves were also drawn using the data in the TCGA Validation set, TCGA Entire set and CGGA set. All results demonstrated a high predictive ability of the immune-related signature. The analysis of the correlations between risk score and survival rate of samples in GBM cohorts or LGG cohorts, which showed the same results (Supplementary Figure S2).

### Immune-Related Signature as an Independent Prognostic Factor

To verify whether the immune-related signature may act as an independent factor to predict the survival of patients with glioma, both univariate and multivariate analyses between risk score, OS and other clinical indicators were carried out (Table 3). Results of the multivariate analysis using the Entire TCGA set and CGGA set demonstrated that risk score, age, histology, tumor grade and 1p19q-co-deletion-status may act as independent prognostic indicators for patients with glioma (*p* < 0.05). As there is no TNM stage in patients with glioma, the category of TNM stage was omitted.

**TABLE 3:** Univariate and multivariate analyses using the Cox proportional hazard model. HR, hazard ratio; CI, confidence interval.

Overall survival	Univariate analysis			Multivariate analysis		
	HR	95%CI	*p*-value	HR	95%CI	*p*-value
Entire TCGA Set						
Risk Score (high vs. low)	2.696	(2.434–2.986)	3.10E-97	2.063	(1.778–2.394)	1.41E-21
Age	1.066	(1.056–1.076)	4.13E-45	1.032	(1.021–1.043)	1.81E-09
Grade	4.682	(3.852–5.690)	5.43E-66	1.514	(1.103–2.076)	0.01
Gender	0.795	(0.618–1.204)	0.075	0.836	(0.652–1.072)	0.158
Histology	0.404	(0.251–0.465)	1.78E-42	0.994	(0.830–1.189)	0.945
CGGA Set						
Risk Score (high vs. low)	12.58	(8.349–18.956)	6.72E-37	6.569	(3.377–12.778)	2.93E-08
Age	1.032	(1.020–1.046)	3.66E-07	1.016	(1.003–1.029)	0.019
Grade	2.627	(2.229–3.095)	5.83E-34	1.642	(1.308–2.060)	1.90E-05
Gender	0.941	(0.716–1.236)	0.65961786	0.882	(0.664–1.173)	0.390
Histology	1.128	(1.079–1.180)	1.08E-07	1.117	(1.039–1.20)	0.003
IDH_mutation_status	0.351	(0.266–0.463)	1.59E-14	1.431	(0.937–2.184)	0.097
1p19q-co-deletion-status	0.29	(0.198–0.428)	4.23E-11	0.477	(0.3–0.760)	0.002
MGMTp_methylation	0.950	(0.771–1.173)	0.637863187	1.132	(0.889–1.441)	0.315

### Immune-Related Signature and Clinicopathological Factors

**FIGURE 3:**
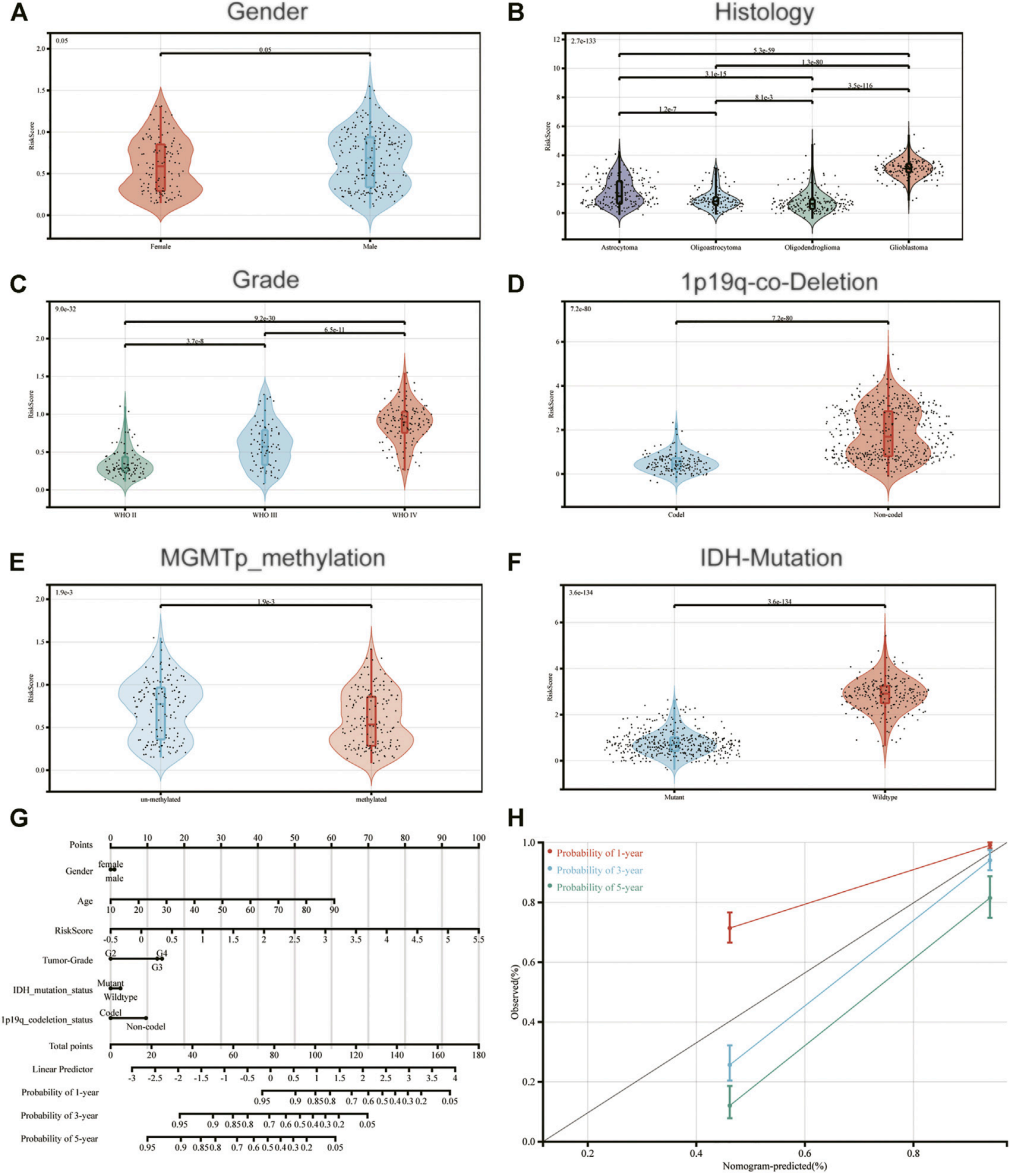
Association between the constructed immune-related signature and **(A)** Gender; **(B)** Histology; **(C)** Grade; **(D)** 1p19q-co-Deletion-Status; **(E)** MGMTp-methylation-status and **(F)** IDH-Mutation-Status. **(G)** A nomogram to predict 1-, 3- and 5-year OS; **(H)** Calibration curves determined using a nomogram to demonstrate the consistency between predicted and detected 1-, 3- and 5-year survival.

To determine the association between IRDEGs prognostic risk score and the other clinicopathological factors, independent t-tests were performed between risk score, gender, histology, tumor grade, 1p19q-co-deletion, MGMTp-methylation and IDH mutation. Risk scores were notably increased in advanced grade tumors (such as glioblastoma), deleted-1p19q, methylated-MGMTp and IDH-wildtype (Figures 3B–F). However, there was no significant difference between males and females for risk scores (Figure 3A).

Moreover, a nomogram was created according to risk score and the aforementioned clinicopathological indicators, for constructing an accurate tool to predict the survival of patients with glioma (Figure 3G). Notably, the calibration curves of the prognostic nomogram suggested a positive consistency between predicted and exact 1-year, 3-year and 5-year survival (Figure 3H).

### Immune-Related Signature and Immune Cell Infiltration

**FIGURE 4:**
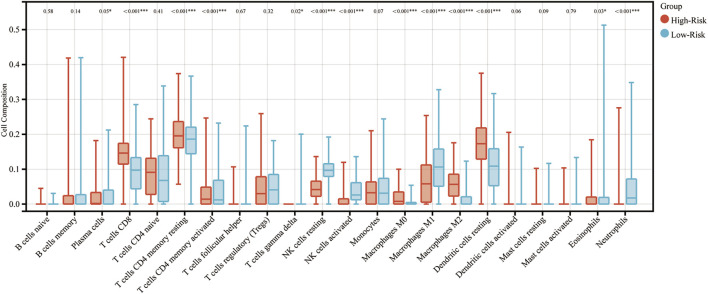
Association between immune cell infiltration and the immune-related signature in glioma. The blue columns represent the low-risk groups. The red columns represent the high-risk groups.

To determine functions of the immune-related signature in the TIME, CIBERSORT was used to estimate immune cell infiltration, and to estimate the relative proportion of 22 types of immune cells in glioma. Immune cell type abundance in the high-risk group and low-risk group are displayed in Table 4. As shown in Figure 4 and Table 4, 5 types of immune cells (T cells CD8, T cells CD4 memory resting, Macrophages M0, Macrophages M2 and Dendritic cells resting) exhibited a positive correlation with risk score (*p* < 0.05; Figure 4; Table 4). In total, 7 types of immune cells (Plasma cells, T cells CD4 memory activated, NK cells resting, NK cells activated, Macrophages M1, Eosinophils and Neutrophils) were negatively correlated with IRDEGs prognostic risk score (*p* < 0.05; Figure 4; Table 4).

**TABLE 4:** 22 Immune cell type abundance between the high-risk and low-risk groups in the entire TCGA set. TCGA, The Cancer Genome Atlas.

Immune cell types	Abundance		Method	*p*-value
	High-Risk (Mean ± std)	Low-Risk (Mean ± std)		
B cells naive	4.4e-4 ± 3.5e-3	3.1e-4±2.7e-3	t-test	0.58
B cells memory	0.02 ± 0.04	0.02 ± 0.06	t-test	0.14
Plasma cells	0.02 ± 0.03	0.02 ± 0.04	t-test	0.05*
T cells CD8	0.14 ± 0.06	0.09 ± 0.06	t-test	< 0.001***
T cells CD4 naive	0.08 ± 0.06	0.08 ± 0.07	t-test	0.41
T cells CD4 memory resting	0.20 ± 0.05	0.18 ± 0.06	t-test	< 0.001***
T cells CD4 memory activated	0.03 ± 0.04	0.04 ± 0.05	t-test	< 0.001***
T cells follicular helper	4.4e-3±0.02	3.7e-3±0.02	t-test	0.67
T cells regulatory (Tregs)	0.05 ± 0.05	0.05 ± 0.05	t-test	0.32
T cells gamma delta	0.0e+0 ± 0.0e+0	2.0e-3±0.02	t-test	0.02*
NK cells resting	0.04 ± 0.03	0.09 ± 0.04	t-test	< 0.001***
NK cells activated	0.01 ± 0.02	0.04 ± 0.03	t-test	< 0.001***
Monocytes	0.04 ± 0.04	0.05 ± 0.05	t-test	0.07
Macrophages M0	0.02 ± 0.02	4.8e-3±9.4e-3	t-test	< 0.001***
Macrophages M1	0.07 ± 0.06	0.11 ± 0.07	t-test	< 0.001***
Macrophages M2	0.06 ± 0.04	0.01 ± 0.02	t-test	< 0.001***
Dendritic cells resting	0.17 ± 0.07	0.11 ± 0.07	t-test	< 0.001***
Dendritic cells activated	0.01 ± 0.03	6.4e-3±0.02	t-test	0.06
Mast cells resting	7.3e-3 ± 0.02	5.2e-3±0.01	t-test	0.09
Mast cells activated	4.5e-3 ± 0.01	4.8e-3±0.02	t-test	0.79
Eosinophils	0.02 ± 0.03	0.02 ± 0.05	t-test	0.03*
Neutrophils	5.0e-3 ± 0.02	0.05 ± 0.06	t-test	< 0.001***

**FIGURE 5:**
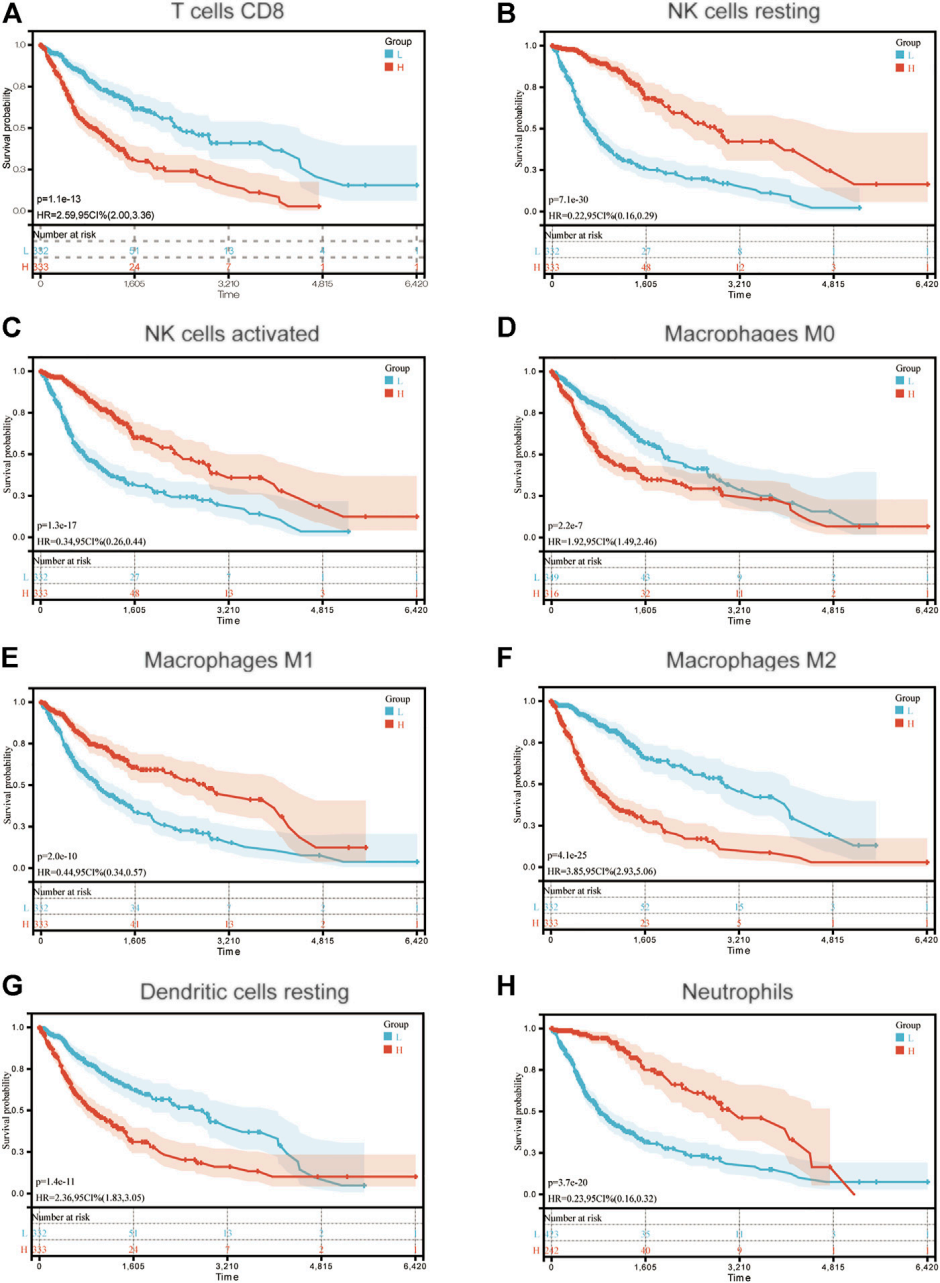
Association between OS and **(A)** T cells CD8; **(B)** NK cells resting; **(C)** NK cells activated; **(D)** Macrophages M0; **(E)** Macrophages M1; **(F)** Macrophages M2; **(G)** Dendritic cells resting and **(H)** Neutrophils in patients with glioma. OS, overall survival.

Moreover, the samples were divided into high and low groups according to the median of different types of immune cell infiltrations, and the K-M survival curves were used to determine the association between the OS and immune cell infiltration. As the risk score demonstrated a negative correlation with glioma prognosis, it was hypothesized that the association between immune cell infiltration and risk score, and between the immune cell infiltration and survival would demonstrate the opposite trends. In total, 8 types of immune cells (T cells CD8, NK cells resting, NK cells activated, Macrophages M0, Macrophages M1, Macrophages M2, Dendritic cells resting and Neutrophils) proved this hypothesis. The K-M survival curves of these are displayed in Figure 5.

### Immune-Related Signature and TMB

**FIGURE 6:**
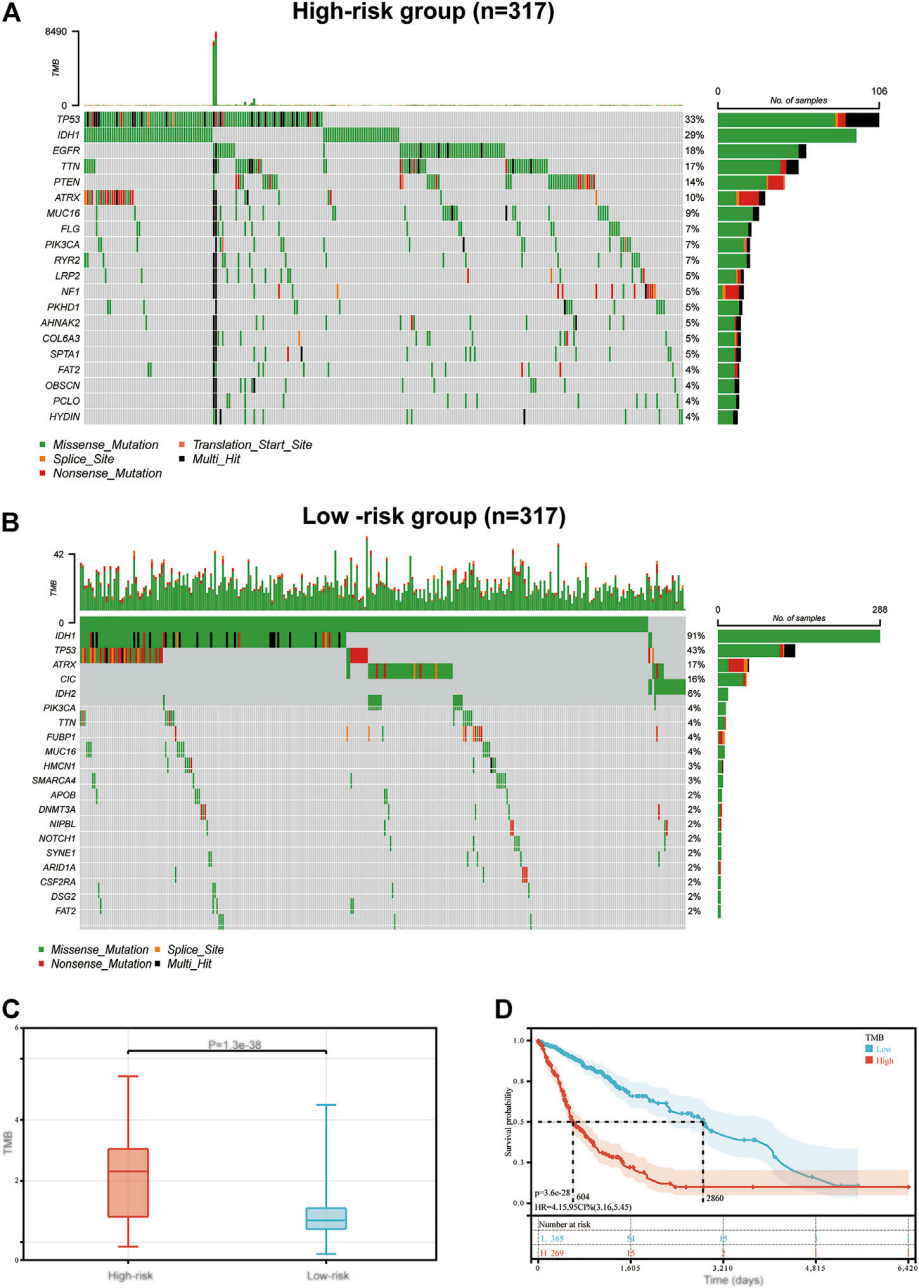
The mutation profile and TMB in low-risk and high-risk groups. Mutation profile in **(A)** high-risk and **(B)** low-risk groups. **(C)** The association between the immune-related signature and TMB. **(D)** The association between TMB and OS in the TCGA GBM and LGG datasets. TMB, tumor mutation burden; OS, overall survival; TCGA, The Cancer Genome Atlas; GBM, glioblastoma multiforme; LGG, low grade glioma.

Results of previous studies have demonstrated that TMB acts as a biomarker of prognosis and immunotherapy (Halbert and Einstein, 2021). Thus, in the present study, the association between immune-related signature and TMB were determined, in order to explain the predictive capability of the immune-related signature. The top 20 most frequently mutated genes in the low-risk and high-risk groups are displayed in Figure 6A and Figure 6B, respectively. Moreover, TMB in the high-risk group was compared with that in the low-risk group. Results of the present study demonstrated that TMB exhibited a positive correlation with risk score (*p* < 0.001; Figure 6C). The samples were further allocated into high-TMB and low-TMB groups, and the OS was compared between these two groups (*p* < 0.001; Figure 6D). Notably, OS in the high-TMB group was decreased compared with that in the low-TMB group.

### Immune-Related Signature and Immune Checkpoint Inhibitors

**FIGURE 7:**
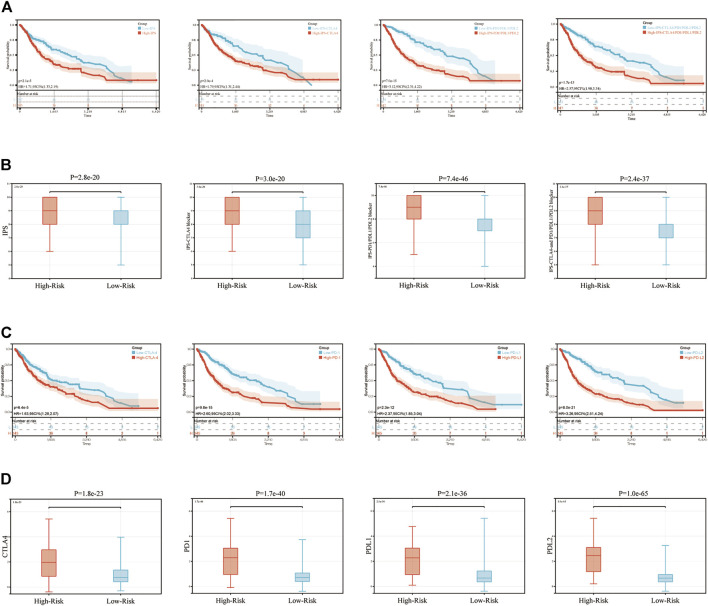
IPS and immunotherapy gene expression analysis. **(A)** The association between OS and IPS, IPS-CTLA4 blocker, IPS-PD1PDL1PDL2 blocker, IPS-CTLA4-and PD1PDL1PDL2 blocker. **(B)** The association between IPS and immune-related signature in patients with glioma. **(C)** The association between OS and CTLA4, PD1, PDL1, PDL2. **(D)** The gene expression of PD1, CTLA4, PD-L1, and PD-L2 in low-risk and high-risk groups. IPS, immunophenoscore.

The present study utilized four types of score (IPS, IPS-CTLA4, IPS-PD1-PDL1-PDL2 and IPS-CTLA4-PD1-PDL1-PDL2) to evaluate the potential of patients with glioma to be placed on ICIs. Firstly, we evaluated the association between these four types of score and OS in glioma. The results showed these four scores significantly had the negative correlation with the OS (*p* < 0.001, Figure 7A). Then these four scores were significantly elevated in the high-risk group compared with those in low-risk group (*p* < 0.001; Figure 7B), which explained the prognostic ability of our signature. Moreover, the same analyses were performed on the expression levels of CTLA4, PD1, PDL1 and PDL2, which were significantly related with the OS of glioma patients and increased in the high-risk group (*p* < 0.001; Figures 7C,D).

## Discussion

Due to the high level of malignancy and poor prognosis of glioma (Xun et al., 2021), the development of accurate methods is required to predict the associated prognosis and to create effective therapy for patients with glioma. At present, immunotherapy demonstrates high levels of effectiveness in the treatment of malignant tumors (Chen et al., 2021b; Sprooten et al., 2021). Results of previous studies have demonstrated that TIME exhibits a high association with the occurrence and development of several tumor types, and this may act as an important prognostic indicator (Berraondo et al., 2016; Elola et al., 2018). With the development of bioinformatics, many signatures have been established, which demonstrate a high capability to predict the prognosis of patients with tumors (Hermansen et al., 2017; Kang et al., 2020). Therefore, a novel and more accurate immune-related signature was created in the present study to predict the prognosis and response to immunotherapy in patients with glioma.

However, the efficiency of immunotherapy in glioma is not significant at present, indicating that a low number of patients can benefit from it (Touat et al., 2020). The reasons for immunotherapy failure are likely to be complex, but appear to be associated with a lack of biomarkers guiding ICB in different patients (Topalian et al., 2015; Lim et al., 2018). Therefore, a novel signature was constructed to predict the survival and response to ICIs in glioma.

**FIGURE 8:**
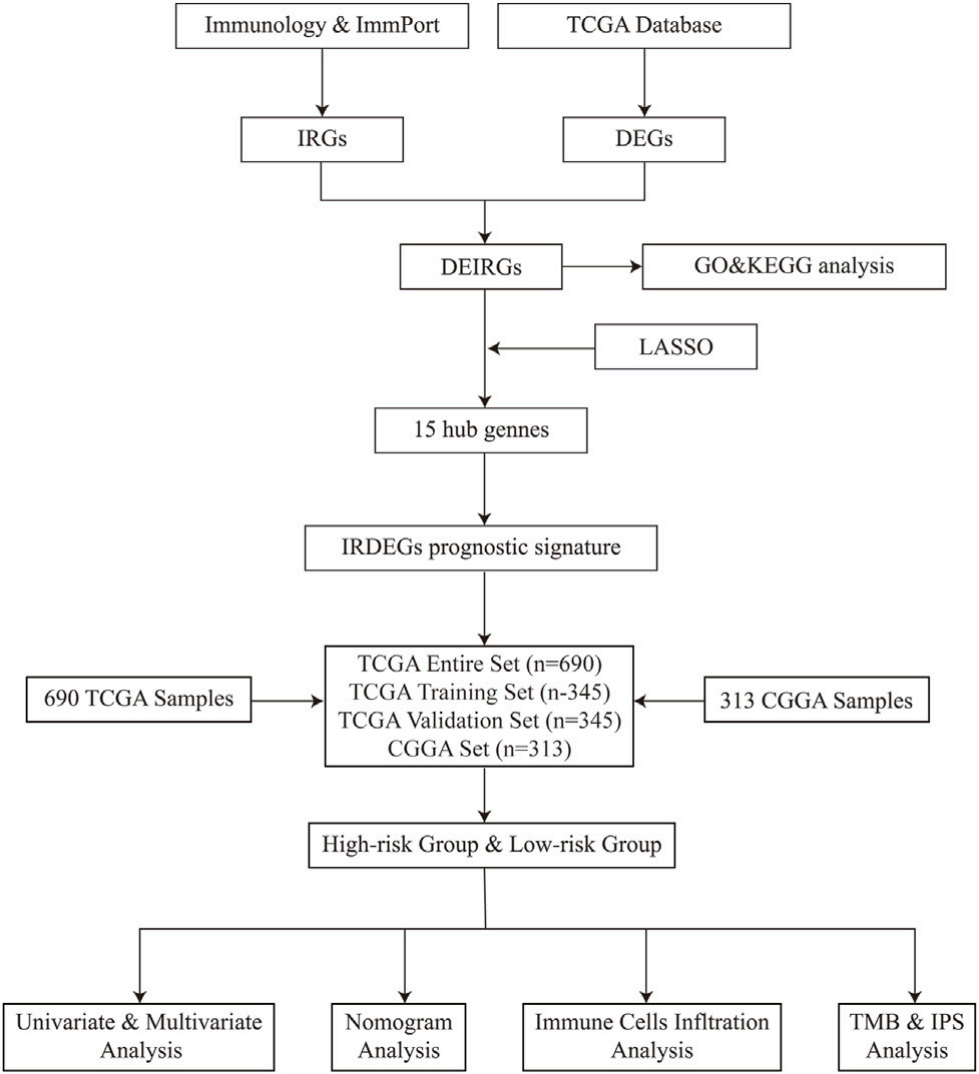
The whole flow gramme.

In the present study, the immune-related signature was constructed using GBM and LGG samples from the TCGA database (Figure 8). In total, 118 genes named DEIRGs were identified to be associated with immune processes and were differently expressed between glioma and healthy tissues. Following the GO and KEGG enrichment analyses, the association between DEIRGs and immune processes were further determined, such as “Antigen processing and presentation” and “T cell receptor signaling pathway”. After performing the Cox analysis and LASSO analysis on 118 DEIRGs, a total of 15 hub genes (MET, SSTR2, CDK4, S100A16, TNFRSF10B, BIRC5, BMP2, ADCYAP1R1, BMP1, APOBEC3C, ANGPTL2, TNFRSF19, VGF, NRG3, and JAG2) were highlighted. Among them, the expression levels of 5 genes (MET, SSTR2, VGF, NRG3 and JAG2) were downregulated, and 10 genes (CDK4, S100A16, TNFRSF10B, BIRC5, BMP2, ADCYAP1R1, BMP1, APOBEC3C, ANGPTL2 and TNFRSF19) were upregulated. Using these 15 hub genes, the immune-related signature was constructed and the risk score of each sample was determined. Samples were allocated into high-risk and low-risk groups according to the median of the risk scores. Moreover, univariate and multivariate analyses were performed using risk score and other clinical indicators. Results of the present study demonstrated that risk score may act as an independent indicator to predict the prognosis of patients with glioma. To verify the predictive accuracy of the constructed signature, the association between risk score and clinicopathological factors were determined, and a nomogram was constructed by integrating risk score and these clinicopathological factors.

To determine the mechanism underlying the predictability of the immune-related signature in the present study, the association between 22 immune cells, risk scores and the prognosis of patients with glioma was determined using CIBERSORT. Results of the present study demonstrated that T cells CD8, Macrophages M0, Macrophages M2 and Dendritic cells resting were negatively associated with the OS of patients with glioma, and NK cells resting, NK cells activated, Macrophages M1 and Neutrophils demonstrated the opposite of these results. Notably, CD8^+^ T cells have previously demonstrated an improved prognosis in the majority of tumor types, which was not demonstrated in the present study. However, this may be due to the immune environment in the CNS. In addition, the association between TMB, risk score and prognosis was determined in patients with glioma. Results of the present study demonstrated that risk score exhibited a positive correlation with TMB, and TMB was negatively associated with the OS of patients with glioma. This may also indicate why the immune-related signature constructed in the present study can predict the prognosis of patients with glioma.

To date, immunotherapy has been used to treat a number of different tumor types, such as lung cancer (Tang et al., 2022), melanoma (Baruch et al., 2021), cervical cancer (Ferrall et al., 2021), liver cancer (Feng et al., 2021). Some immune checkpoints, such as PD-1 and CTLA-4 may lead to anti-tumor immunity (Lee and Seong, 2021). Results of a previous study demonstrated that nivolumab and pembrolizumab, two therapeutics against PD-L1/PD1, have been approved for subsequent line therapy (Leone et al., 2021). However, according to the Phase III clinical trial, the efficiency of immunotherapy in glioma is not significant (Wu and Lim, 2021). These results may still indicate which patients with glioma are suitable for undertaking immunotherapy. Moreover, results of previous studies have demonstrated that TIME and TMB are associated with prognosis in many tumor types, and this is associated with the efficiency of immunotherapy (Cheng et al., 2021; Wei et al., 2021). These results led to the development of the signature in the present study. IPS is an accurate method to predict the response to ICIs, and this has been widely used to guide immunotherapy in numerous tumor types (Meng et al., 2021; Xu et al., 2021). Thus, the association between IPS and risk score was determined in the present study. The results suggested that patients in the high-risk group exhibited increased levels of IPS, which meant that they may exhibit a poor prognosis, but their response to immunotherapy may be improved. Finally, the association between risk score and the other 6 ICBs (LAG3, TIGIT, TIM3, CD27, ICOS, and IDO1), which were predicted by CIBERSORT, was determined. Results of the present study demonstrated an improved immune response compared with traditional PDL1/CTLA4 immune checkpoints in patients with glioma.

In the present study, the accuracy signature was used in a large number of samples, which highlighted the reliability of the signature. Moreover, the predictive mechanism of the signature was determined from several perspectives, including the association between risk score with immune cell infiltration, TMB and IPS. However, the present study exhibits a number of limitations. An increased number of patients with glioma are required to participate in clinical trials to further validate the predictive capability of prognosis and the efficiency of immunotherapy of this signature.

In conclusion, an immune-related signature was constructed in the present study that can accurately predict prognosis and determine the response to immunotherapy in patients with glioma. The constructed signature may provide a deeper theoretical basis, and increase the accuracy of immunotherapy for patients with glioma.

## Data Availability

The datasets presented in this study can be found in online repositories. The names of the repository/repositories and accession number(s) can be found in the article/Supplementary Material.

## Supplementary Material

Supplementary Figure S1 LASSO coefficient profiles and the tuning parameter. **(A)** LASSO coefficient profiles of the 15 hub genes. **(B)** The selection of the tuning parameter (lambda) in the LASSO model by tenfold cross-validation based on minimum criteria for OS.

Supplementary Figure S2 K-M curve analyses between risk score and survival rate in **(A)** TCGA-GBM cohort, **(B)** TCGA-LGG cohort, **(C)** CGGA-GBM cohort, **(D)** CGGA-LGG cohort.

Supplementary Table S1 Totally 118 genes, which were extracted from DEIRGs, showed significant relation with the overall survival (OS) of the samples in the training set by a univariate Cox proportional hazard regression analysis.
