# Corrigendum: Developing an immune-related signature for predicting survival rate and the response to immune checkpoint inhibitors in patients with glioma

**DOI:** 10.3389/fgene.2022.961153

**Published:** 2022-08-17

**Authors:** Sibin Zhang, Xu Xiao, Yu Wang, Tianjun Song, Chenlong Li, Hongbo Bao, Qing Liu, Guiyin Sun, Xiaoyang Sun, Tianqi Su, Tianjiao Fu, Yujie Wang, Peng Liang

**Affiliations:** ^1^ Department of Neurosurgery, Harbin Medical University Cancer Hospital, Harbin, China; ^2^ Department of Esophageal Surgery, Harbin Medical University Cancer Hospital, Harbin, China; ^3^ Department of Medicine II, University Hospital LMU Munich, Munich, Germany

**Keywords:** glioma, bioinformatics signature, prognosis, immune enviroment, immunetherapy

In the published article, there was an error in Figure 2 as published. The second ROC curve in Figure 2B and the second KM curve in Figure 2C are same with the first ROC curve in Figure 2B and the first KM curve in Figure 2C, respectively. The corrected Figure 2 appears below.

**FIGURE 2:**
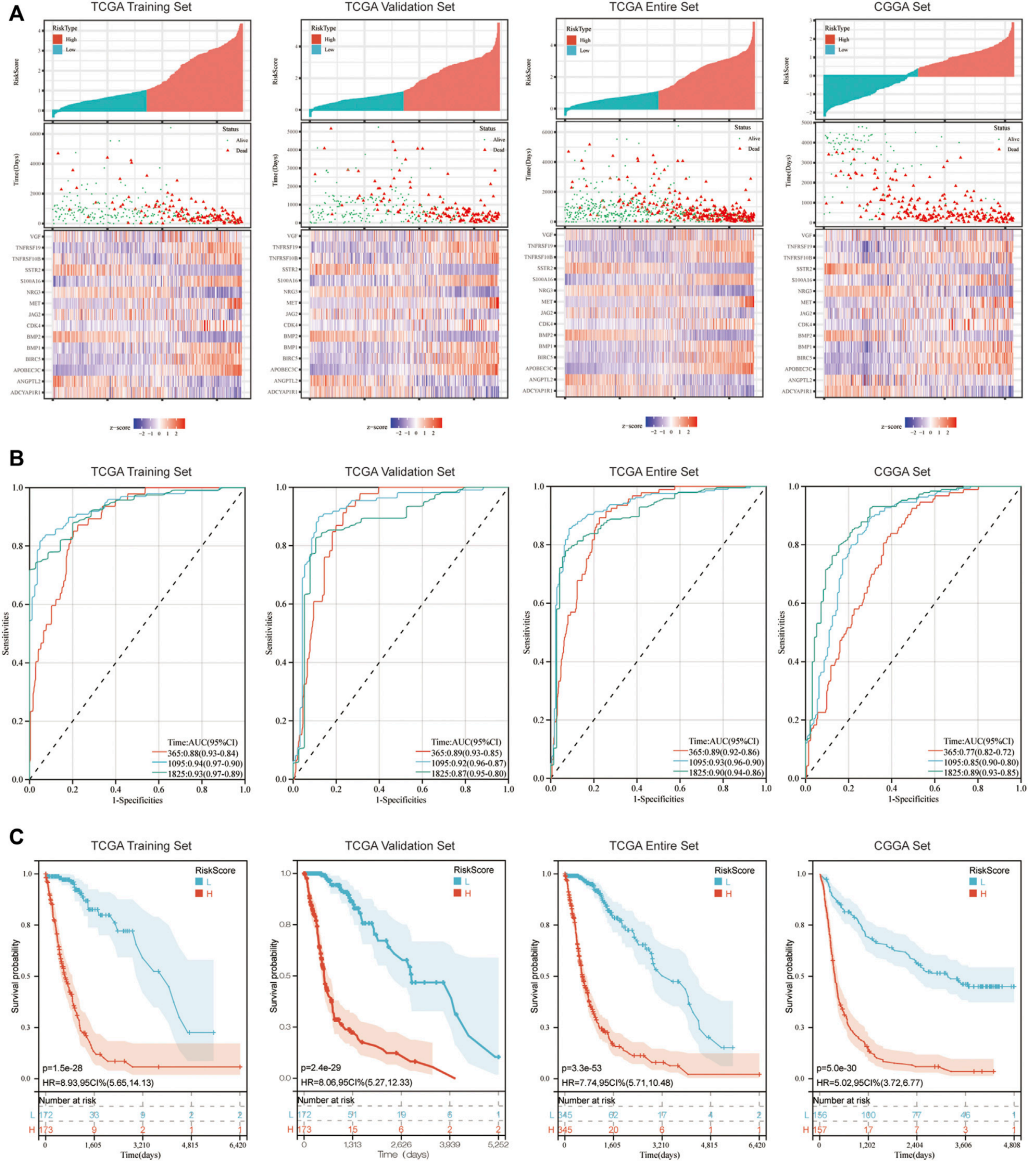
Immune-related signature effectively predicts the prognosis of patients with glioma. **(A)** Risk score distribution, survival status and expression of 15 hub genes for glioma in low-risk and high-risk groups. **(B)** 1, 3 and 5-year ROC curve analyses. ROC, receiver operating characteristic. **(C)** K-M survival curve analyses.

In the published article, there was an error in **Results**, *Immune-Related Signature and the Survival of Patients With Glioma*, **paragraph 1**. This sentence previously stated: “In the TCGA training set, the under areas of 1-year, 3-year and 5-year survival were 0.88, 0.93 and 0.90”

The corrected sentence appears below:

“In the TCGA training set, the under areas of 1-year, 3-year and 5-year survival were 0.88, 0.94 and 0.93”

The authors apologize for this error and state that this does not change the scientific conclusions of the article in any way. The original article has been updated.

