# Supplementary material for: Developing an Immune-Related Signature for Predicting Survival Rate and the Response to Immune Checkpoint Inhibitors in Patients With Glioma
Source: Front Genet. 2022 Jun 2;13:899125. doi: 10.3389/fgene.2022.899125 (PMC9204856; doi:10.3389/fgene.2022.899125)
Supplement: Supplementary file 1 [file Table1.docx]

| Tag | p.value | HR | Low 95%CI | High 95%CI |
| --- | --- | --- | --- | --- |
| MET | 2.69E-10 | 1.812564878 | 1.507084464 | 2.179965036 |
| CMTM6 | 5.23E-19 | 3.484791499 | 2.647739598 | 4.586467566 |
| PCSK2 | 1.17E-06 | 0.688035358 | 0.591747371 | 0.799991141 |
| SSTR2 | 2.04E-18 | 0.408161892 | 0.3339717 | 0.498833074 |
| HLA-DQA2 | 4.24E-08 | 1.346972346 | 1.210870119 | 1.498372511 |
| CORT | 0.00184516 | 0.544940289 | 0.371886516 | 0.79852295 |
| CDK4 | 5.67E-13 | 1.456316635 | 1.314818097 | 1.61304301 |
| S100A16 | 1.91E-17 | 2.345562848 | 1.926914279 | 2.855168565 |
| PTN | 5.85E-08 | 1.533615209 | 1.314006517 | 1.789926899 |
| B2M | 7.31E-14 | 2.094800153 | 1.725917115 | 2.542525155 |
| CD320 | 1.71E-07 | 2.159831469 | 1.6182618 | 2.88264357 |
| TRIM5 | 3.25E-14 | 2.645708077 | 2.057776014 | 3.401619602 |
| HTR1A | 5.93E-08 | 0.361453019 | 0.250187521 | 0.522201446 |
| LANCL1 | 2.01E-08 | 0.382607544 | 0.273539981 | 0.535163202 |
| PPP4C | 3.86E-18 | 4.941968039 | 3.445618031 | 7.088147287 |
| TAFA2 | 1.99E-10 | 0.352716035 | 0.255859423 | 0.486238106 |
| NMB | 0.013592393 | 0.875128099 | 0.787163844 | 0.972922213 |
| GPI | 3.97E-08 | 2.034012725 | 1.578748462 | 2.620561708 |
| TNFRSF10B | 5.65E-10 | 2.089758605 | 1.655414225 | 2.638065423 |
| BIRC5 | 5.65E-18 | 1.669438166 | 1.486201132 | 1.8752669 |
| MAPK3 | 3.15E-05 | 0.449891532 | 0.308867382 | 0.655305163 |
| PSMB8 | 2.27E-13 | 2.138305643 | 1.745151528 | 2.620030954 |
| PRKCB | 2.88E-10 | 0.545283526 | 0.451594818 | 0.658409071 |
| RASGRP1 | 0.001003953 | 0.702532113 | 0.569251066 | 0.867018789 |
| ACKR3 | 0.0001792 | 1.57559901 | 1.242102663 | 1.998636921 |
| HLA-C | 1.35E-12 | 1.8805972 | 1.579301262 | 2.239373774 |
| FCER1G | 1.59E-12 | 1.805851371 | 1.532796639 | 2.127548489 |
| CHGB | 3.37E-17 | 0.648340961 | 0.586224229 | 0.717039626 |
| BMP2 | 3.24E-22 | 0.49850682 | 0.433048032 | 0.573860244 |
| VIPR1 | 0.000354813 | 0.428791424 | 0.269423755 | 0.68242715 |
| PDIA2 | 1.92E-09 | 0.545321683 | 0.447388958 | 0.664691725 |
| JAG1 | 5.82E-16 | 1.96394336 | 1.66778037 | 2.312698717 |
| TNFSF13B | 0.000768788 | 1.277801983 | 1.107722578 | 1.47399533 |
| VIP | 0.000991063 | 0.627358815 | 0.475336577 | 0.828000835 |
| THRB | 1.07E-05 | 0.550256734 | 0.421737917 | 0.717939891 |
| S100A10 | 7.79E-13 | 1.630461468 | 1.426370735 | 1.863754306 |
| PPP3CA | 3.36E-09 | 0.382196521 | 0.277858019 | 0.525715187 |
| ZC3HAV1 | 2.03E-05 | 2.139241518 | 1.507854413 | 3.035010697 |
| CXCL16 | 8.34E-06 | 1.789048225 | 1.385211984 | 2.310616418 |
| ADCYAP1R1 | 6.03E-14 | 0.653592136 | 0.584910507 | 0.73033853 |
| PLTP | 4.35E-05 | 1.568239918 | 1.263939423 | 1.945802461 |
| GNAI1 | 9.26E-20 | 0.45372641 | 0.382694151 | 0.53794304 |
| BMP1 | 2.18E-15 | 2.534604562 | 2.014119598 | 3.189592264 |
| PDGFRA | 4.79E-08 | 0.714684057 | 0.633481794 | 0.806295154 |
| APOBEC3C | 6.16E-27 | 2.654331514 | 2.22143472 | 3.171588039 |
| CTSS | 1.14E-11 | 1.806299688 | 1.522810495 | 2.142563748 |
| ANGPTL2 | 1.76E-16 | 0.557696714 | 0.48535298 | 0.64082356 |
| SEMA6B | 1.88E-18 | 0.512609672 | 0.441452431 | 0.595236672 |
| TNC | 4.43E-14 | 1.569724316 | 1.396277991 | 1.764716228 |
| PMP2 | 0.007608644 | 0.866359934 | 0.779734674 | 0.962608898 |
| SEMA4D | 1.01E-07 | 0.488044967 | 0.37478713 | 0.635528465 |
| HLA-A | 1.32E-14 | 2.166326915 | 1.779562366 | 2.637149668 |
| PROCR | 1.84E-14 | 1.956973341 | 1.64812676 | 2.323695452 |
| CRHR1 | 1.88E-10 | 0.315052881 | 0.220827513 | 0.449483476 |
| SST | 0.002798983 | 0.840895641 | 0.750573895 | 0.942086426 |
| TAFA1 | 5.70E-07 | 0.513798791 | 0.395779737 | 0.667010391 |
| CMTM3 | 1.86E-18 | 4.167036758 | 3.028521368 | 5.733555497 |
| SEMA4A | 1.05E-10 | 0.386766214 | 0.289919726 | 0.515963871 |
| HLA-DMA | 7.89E-11 | 1.699023496 | 1.448154421 | 1.993351536 |
| ADRB1 | 0.004003217 | 0.621941237 | 0.450074992 | 0.85943656 |
| PLAAT4 | 8.20E-06 | 1.49195588 | 1.2514048 | 1.77874685 |
| EGFR | 6.26E-06 | 1.278782484 | 1.149371228 | 1.422764553 |
| SLC40A1 | 3.27E-07 | 1.618492881 | 1.345418193 | 1.946992556 |
| VCAM1 | 1.09E-05 | 1.308293227 | 1.160649989 | 1.474717775 |
| MMP9 | 2.41E-18 | 1.462647001 | 1.343044825 | 1.59290011 |
| HLA-B | 3.51E-13 | 1.809044835 | 1.541959878 | 2.122391939 |
| CD22 | 0.001847645 | 0.615122519 | 0.453022674 | 0.835224669 |
| FGF13 | 3.52E-11 | 0.299077706 | 0.209244951 | 0.427477338 |
| TNFRSF19 | 1.59E-18 | 2.142164531 | 1.807265463 | 2.539122764 |
| PAK3 | 4.47E-13 | 0.450917277 | 0.363465928 | 0.559409768 |
| CSRP1 | 0.001196789 | 1.28602181 | 1.104469669 | 1.497417397 |
| TRIM22 | 5.96E-11 | 1.783050115 | 1.499498166 | 2.120221141 |
| CCN2 | 0.009318203 | 1.210306232 | 1.048117158 | 1.397592973 |
| GRN | 1.21E-13 | 2.944554687 | 2.213374463 | 3.917277645 |
| VIM | 8.70E-17 | 1.624463237 | 1.449040325 | 1.8211231 |
| LCNL1 | 9.89E-12 | 0.511023605 | 0.421215865 | 0.619979319 |
| MSR1 | 8.91E-22 | 2.122523015 | 1.819881579 | 2.475492911 |
| A2M | 0.002661039 | 1.370209485 | 1.115711853 | 1.682758883 |
| GREM2 | 0.007884282 | 0.620571848 | 0.43645767 | 0.882352275 |
| SSTR1 | 3.68E-16 | 0.423570371 | 0.34450308 | 0.520784486 |
| HLA-DMB | 1.60E-13 | 1.882332774 | 1.591225995 | 2.226696071 |
| TYROBP | 1.33E-08 | 1.649252258 | 1.38783548 | 1.959910271 |
| APOM | 0.000290194 | 0.463090728 | 0.305382157 | 0.702244768 |
| PAK5 | 1.81E-18 | 0.326510178 | 0.25423898 | 0.419325535 |
| MASP1 | 0.001752752 | 0.782473497 | 0.671034697 | 0.91241895 |
| RFXANK | 0.000128156 | 2.086908564 | 1.432186821 | 3.040935227 |
| ELN | 2.16E-13 | 1.67261248 | 1.457904909 | 1.918940316 |
| ARRB1 | 1.19E-11 | 0.451604094 | 0.358900393 | 0.568253092 |
| FGF12 | 8.88E-18 | 0.504158268 | 0.431203965 | 0.589455525 |
| VGF | 0.009106473 | 1.115909755 | 1.027626511 | 1.211777398 |
| NOS1 | 0.000145349 | 0.442719092 | 0.290773389 | 0.674065103 |
| S100A6 | 1.21E-21 | 1.947088973 | 1.698402013 | 2.232189695 |
| NFKBIA | 0.001599502 | 0.693455362 | 0.552441759 | 0.870463413 |
| PTGDS | 4.65E-08 | 0.804469505 | 0.744076903 | 0.869763841 |
| PGF | 0.018428846 | 0.82867026 | 0.708775713 | 0.968845837 |
| PRKCG | 6.78E-05 | 0.718141923 | 0.610195257 | 0.845184908 |
| PCSK1 | 0.021580143 | 1.162613104 | 1.022392597 | 1.322064768 |
| NPY | 0.001953137 | 0.825088384 | 0.73056963 | 0.931835669 |
| FCGRT | 5.87E-11 | 2.600239446 | 1.953317762 | 3.461415908 |
| PPP3CB | 4.56E-18 | 0.229701791 | 0.164681361 | 0.320393956 |
| TAP1 | 1.96E-11 | 1.872616689 | 1.559025728 | 2.24928505 |
| F2R | 3.55E-06 | 1.631514352 | 1.326543954 | 2.00659697 |
| AMH | 1.51E-05 | 0.708432461 | 0.606013644 | 0.828160481 |
| NRG3 | 5.64E-22 | 0.406623134 | 0.338610921 | 0.488296044 |
| IL17RD | 6.23E-07 | 0.502871299 | 0.383754518 | 0.658961736 |
| CALCRL | 2.10E-11 | 0.657290169 | 0.581347032 | 0.74315399 |
| JAG2 | 0.000345252 | 0.480008092 | 0.321128225 | 0.717494603 |
| GBP2 | 8.81E-07 | 1.3801635 | 1.213797596 | 1.5693319 |
| IL6 | 3.11E-05 | 1.302473203 | 1.150186205 | 1.47492331 |
| CXCR4 | 2.62E-12 | 1.708927378 | 1.470723185 | 1.985712071 |
| CXCL14 | 1.24E-06 | 1.28326925 | 1.160208404 | 1.419382898 |
| CRABP2 | 0.001182694 | 1.204418254 | 1.076362236 | 1.347709239 |
| IL10RB | 7.75E-18 | 3.991054753 | 2.911719506 | 5.470485055 |
| CKLF | 1.44E-18 | 3.159355588 | 2.444885105 | 4.08261628 |
| WNT5A | 0.000208414 | 1.493133407 | 1.208060775 | 1.845476169 |
| EDNRB | 3.76E-05 | 0.793181098 | 0.710434263 | 0.885565754 |
| MDK | 2.74E-13 | 1.440071315 | 1.305861157 | 1.58807495 |
| CHGA | 1.70E-11 | 0.703587916 | 0.635115769 | 0.77944208 |

Table S1. Totally 118 genes, which were extracted from DEIRGs, showed significant relation with the overall survival (OS) of the samples in the training set by a univariate Cox proportional hazard regression analysis. The 15 hub genes were highlighted by red color.
