# Supplementary figures and images for: Developing an Immune-Related Signature for Predicting Survival Rate and the Response to Immune Checkpoint Inhibitors in Patients With Glioma
Source: Front Genet. 2022 Jun 2;13:899125. doi: 10.3389/fgene.2022.899125 (PMC9204856; doi:10.3389/fgene.2022.899125)

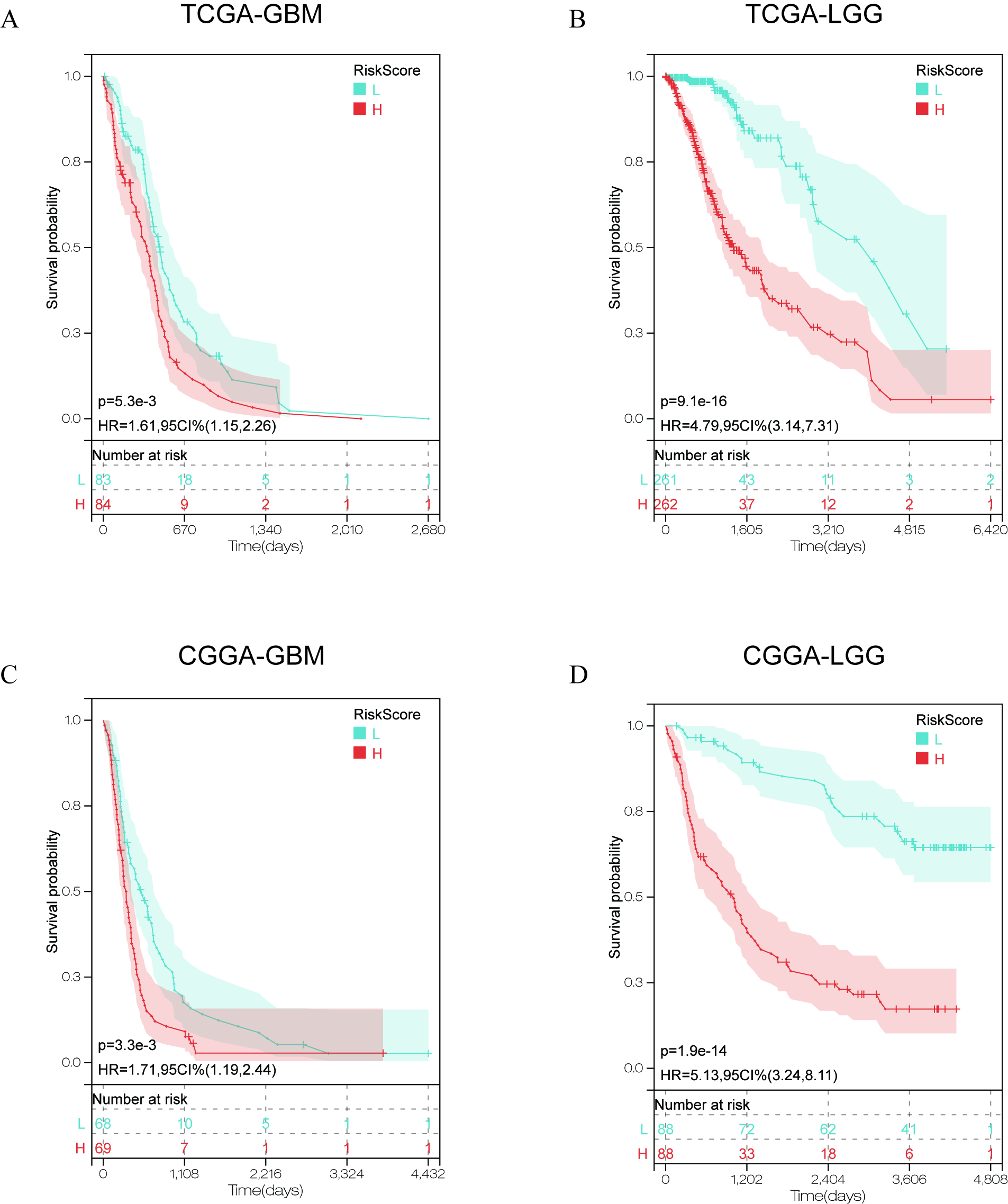

Supplement: Supplementary file 2 [file Image2.TIF]

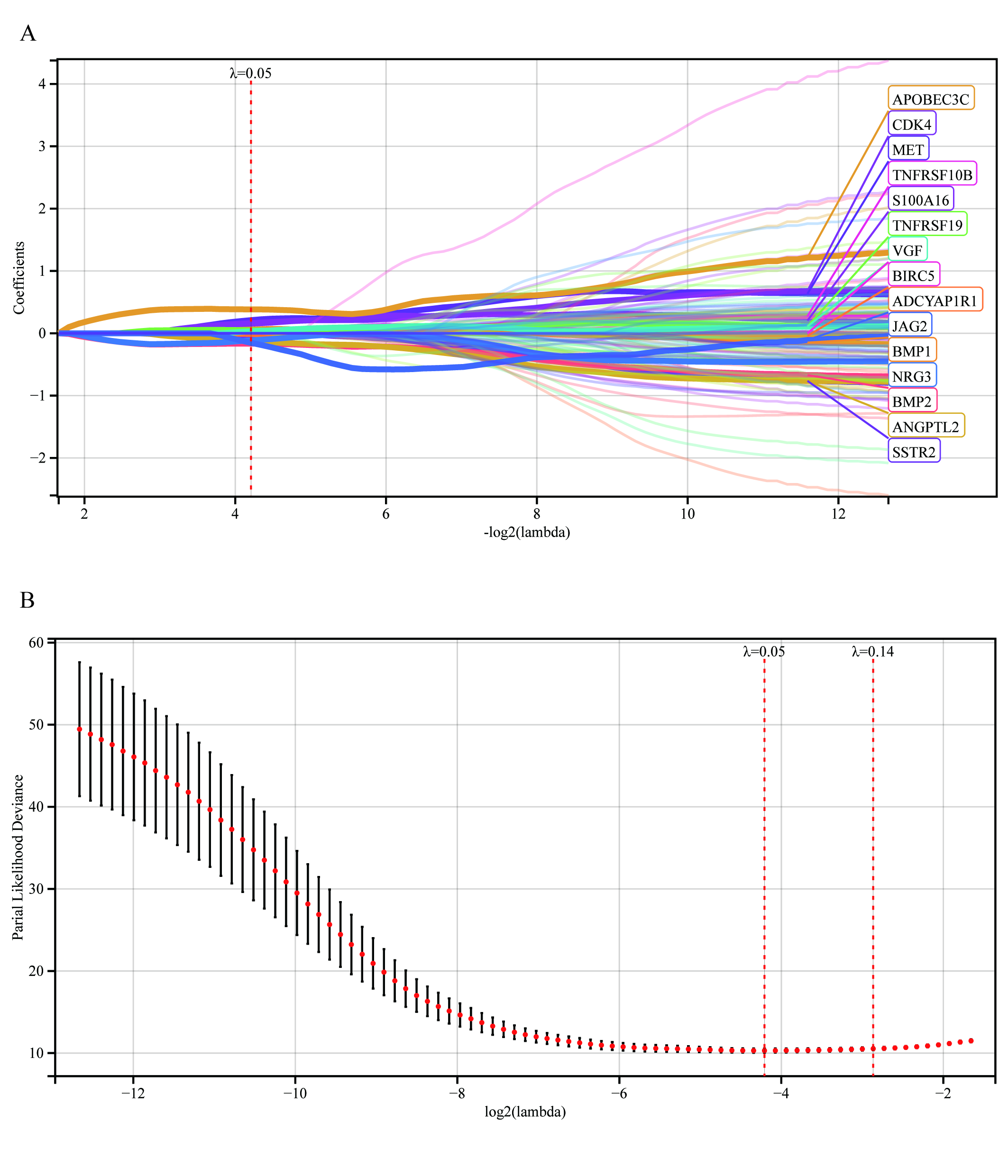

Supplement: Supplementary file 3 [file Image1.TIF]
